# Charge Density Analysis of Actinide Compounds from the Quantum Theory of Atoms in Molecules and Crystals

**DOI:** 10.1021/acs.jpclett.1c00100

**Published:** 2021-02-12

**Authors:** Alessandro Cossard, Jacques K. Desmarais, Silvia Casassa, Carlo Gatti, Alessandro Erba

**Affiliations:** † Dipartimento di Chimica, Università di Torino, via Giuria 5, 10125 Torino, Italy; ‡ CNR-SCITEC, Istituto di Scienze e Tecnologie Chimiche “Giulio Natta”, via C. Golgi 19, 20133 Milano, Italy

## Abstract

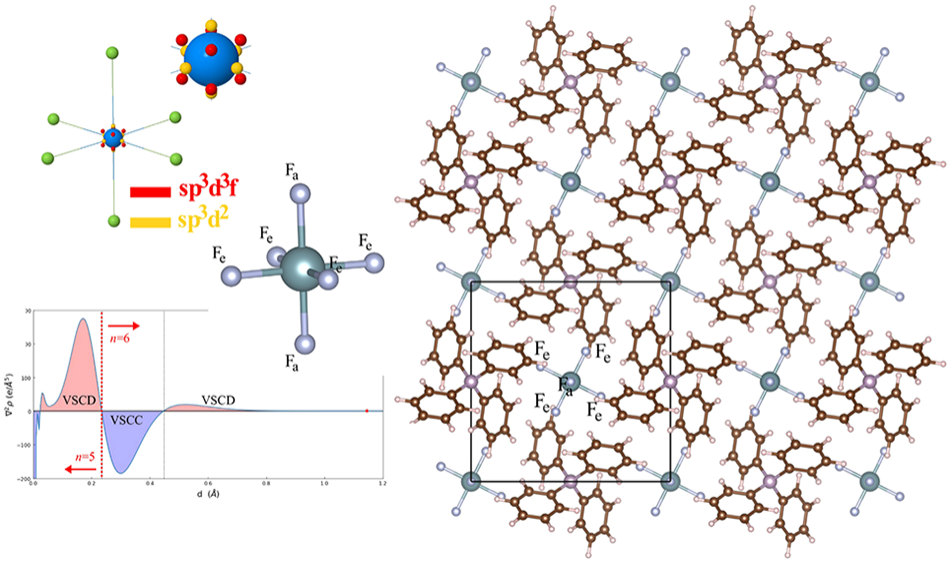

The nature of chemical bonding in actinide compounds (molecular complexes and materials) remains elusive in many respects. A thorough analysis of their electron charge distribution can prove decisive in elucidating bonding trends and oxidation states along the series. However, the accurate determination and robust analysis of the charge density of actinide compounds pose several challenges from both experimental and theoretical perspectives. Significant advances have recently been made on the experimental reconstruction and topological analysis of the charge density of actinide materials [Gianopoulos *et al*. IUCrJ, 2019, 6, 895]. Here, we discuss complementary advances on the theoretical side, which allow for the accurate determination of the charge density of actinide materials from quantum-mechanical simulations in the bulk. In particular, the extension of the Topond software implementing Bader’s quantum theory of atoms in molecules and crystals (QTAIMAC) to *f*- and *g*-type basis functions is introduced, which allows for an effective study of lanthanides and actinides in the bulk and *in vacuo*, on the same grounds. Chemical bonding of the tetraphenyl phosphate uranium hexafluoride cocrystal [PPh_4_^+^][UF_6_^–^] is investigated, whose experimental charge density is available for comparison. Crystal packing effects on the charge density and chemical bonding are quantified and discussed. The methodology presented here allows reproducing all subtle features of the topology of the Laplacian of the experimental charge density. Such a remarkable qualitative and quantitative agreement represents a strong mutual validation of both approaches—experimental and computational—for charge density analysis of actinide compounds.

Chemical bonding in actinide compounds is a complex and fascinating phenomenon, yet to be fully rationalized, with both fundamental and technological implications. Strong relativistic effects, strong electron correlation, and weak crystal fields contribute to the identification of a broad active valence manifold constituted by the 5*f*, 6*p*, 6*d*, and 7*s* orbital shells, whose degree of participation in the formation of chemical bonds varies as a function of several factors and along the actinide series.^1−4^ In particular, the 5*f* electrons are known to participate in bonding from thorium up to plutonium and then to abruptly become less involved from americium on.^5,6^ An intriguing, much investigated, but still elusive, aspect of actinide chemistry is the occurrence and degree of covalency of 5*f* electrons in the chemical bonding.^1,7−9^ Beside such fundamental aspects, a detailed understanding of chemical bonding in actinide compounds is also relevant to technological applications in the nuclear power industry. In energy production from nuclear fission, the effectiveness of the separation process of uranium from lanthanides and other minor actinides depends on their relative bond strength.^10,11^

A variety of techniques can be used to characterize chemical bonding in actinide compounds, both experimentally (photoelectron, Mössbauer, and X-ray absorption spectroscopies;^9,12−14^ nuclear magnetic resonance;^15^ resonant inelastic X-ray scattering;^6^ and others) and theoretically (energy decomposition analysis;^16,17^ molecular orbital population and bond order analyses;^18−20^ Hirshfeld, Voronoi deformation density, natural bond orbital, and electron localization function analyses;^21−23^ and others). The performance of different theoretical approaches has been recently reviewed.^24−26^

Arguably, the most general, formally rigorous technique allowing for a consistent and quantitative description of multiple aspects of chemical bonding is represented by Bader’s quantum theory of atoms in molecules and crystals (QTAIMAC).^27,28^ At the core of this methodology is the topology of the electron density, and therefore, it can in principle be adopted both experimentally and theoretically, thus allowing for a mutual validation of the two approaches. Despite a broad consensus on its ability to describe subtle features of the chemical bonding, only very recently could the QTAIMAC be successfully applied to actinide compounds because of the many experimental and theoretical challenges related to an accurate determination of their charge density.

Pioneering synchrotron X-ray diffraction measurements on actinide materials with the experimental reconstruction of the electron density date back to the late 1990s.^29,30^ Pinkerton and co-workers have recently reported significant advances in the experimental reconstruction of the charge density of actinide compounds from X-ray diffraction by means of improvements in (i) data collection and reduction strategy and (ii) flexibility of the Hansen–Coppens multipolar formalism.^31−33^ Their improved protocol allowed for the reconstruction of the charge density (and its topological analysis via the QTAIMAC) of the tetraphenyl phosphate uranium hexafluoride cocrystal [PPh_4_^+^][UF_6_^–^].^31^ The accuracy of such an experimental procedure can be evaluated from a comparison with the outcomes of quantum-mechanical simulations.

However, the accurate description of the charge density of actinide compounds is challenging also from a theoretical perspective as one needs to (i) account for relativistic effects, (ii) consider strong electron correlation, (iii) describe the correct localization/delocalization of 5*f* and 6*d* orbitals, and (iv) provide enough variational freedom through a rich and angularly flexible basis set. Recently, the QTAIMAC started being applied to the quantum-mechanical study of chemical bonding in molecular actinide complexes.^34−41^ In particular, Gianopoulos *et al*.^31,32^ computed the charge density of the UF_6_^–^ molecular fragment extracted from the [PPh_4_^+^][UF_6_^–^] crystal, performed a QTAIMAC study, and compared their theoretical results with those from the experiment on the crystals. While an overall agreement between the molecular calculations and the experiments on the crystal was observed for some features of the chemical bonding, some significant quantitative, and even qualitative, discrepancies remained, which require further analysis. In particular, the different topology of the Laplacian of the density around the uranium atom from theory and experiment prevented a full validation of the experimental procedure. The discrepancies were tentatively attributed to missing crystal field effects on the molecular calculations and to the shape of the effective-core pseudopotentials used in the calculations. The former of such effects (*i.e.*, that of the environment on chemical bonding features of actinide complexes) has been the subject of a recent investigation by Wellington and coauthors where, by treating intermolecular interactions with different approaches, it was concluded that it is minor and could not explain the large reported differences between molecular calculations and experiments on the description of U–O bonds in Cs_2_UO_2_Cl_4_, for instance.^42^

In this Letter, we report on both formal and software advances that allowed us to set up a robust computational strategy for the accurate investigation of chemical bonding on both actinide complexes and actinide materials through the QTAIMAC. We have applied our newly developed methodology to the study of chemical bonding on both UF_6_ molecular fragments (both symmetric and distorted, both neutral and charged) and [PPh_4_^+^][UF_6_^–^] crystals. This analysis makes it possible to decouple crystal field effects from intramolecular features of chemical bonding. In particular, the increase of the anisotropy of the charge density distribution, due to the crystal field, around the two sets of nonequivalent fluorine atoms (four equatorial and two apical) bound to the uranium center could be quantified. Crucially, our method describes topological features of the Laplacian of the density around the uranium atom in remarkable agreement with experiment, which strongly validates both approaches.

In our methodology, both molecular and crystalline orbitals are expressed as linear combinations of atomic orbitals (LCAO), which is a suitable representation when chemical features of bonding are to be analyzed. Quantum-mechanical calculations are performed with a developmental version of the Crystal program,^43,44^ where the LCAO approach has recently been extended to *g*-type basis functions.^45,46^ Scalar relativistic effects must be accounted for^39−41,47^ and here are described by use of small-core effective pseudopotentials (with 60 electrons in the core for U).^48,49^ While the program has recently been extended to the treatment of spin–orbit coupling,^50−53^ this relativistic effect is disregarded here. This is because, while making the calculations significantly more demanding, it has been previously shown to induce very minor changes to chemical bonding.^54^ The topological analysis of the electron density ρ(**r**) and of its Laplacian ∇^2^ρ(**r**) is performed with a developmental version of the Topond program^28,55,56^ that was previously parallelized^57^ and that we have here generalized to work in terms of *f*- and *g*-type basis functions, thus allowing for a QTAIMAC analysis of lanthanides and actinides.

Crystals of [PPh_4_^+^][UF_6_^–^] belong to the tetragonal *I*4̅ space group; its UF_6_^–^ molecular subunits are distorted with four equivalent equatorial fluorine atoms and two slightly more elongated apical fluorine atoms (see Figure 1). This species, fully embedded in the crystal lattice, is here labeled cry-UF_6_^–^ (these are calculations performed on the actual periodic structure of the crystal, thus including all PPh_4_^+^ molecules). We have also studied the properties of the distorted, asymmetrical, unit as extracted from the crystal and treated instead as an isolated molecular fragment (a-UF_6_^–^). Calculations have also been performed on a symmetric model of the UF_6_ molecule, both neutral and charged (s-UF_6_ and s-UF_6_^–^). All structural models have been fully relaxed through geometry optimizations. Experimental geometries have also been used for a more direct comparison with experiments. All results presented in the main body of this Letter are obtained with the hybrid B3LYP exchange–correlation functional of the density functional theory (DFT) and basis set BSA (fully uncontracted for the U atom) described in the Supporting Information.

**Figure 1:**
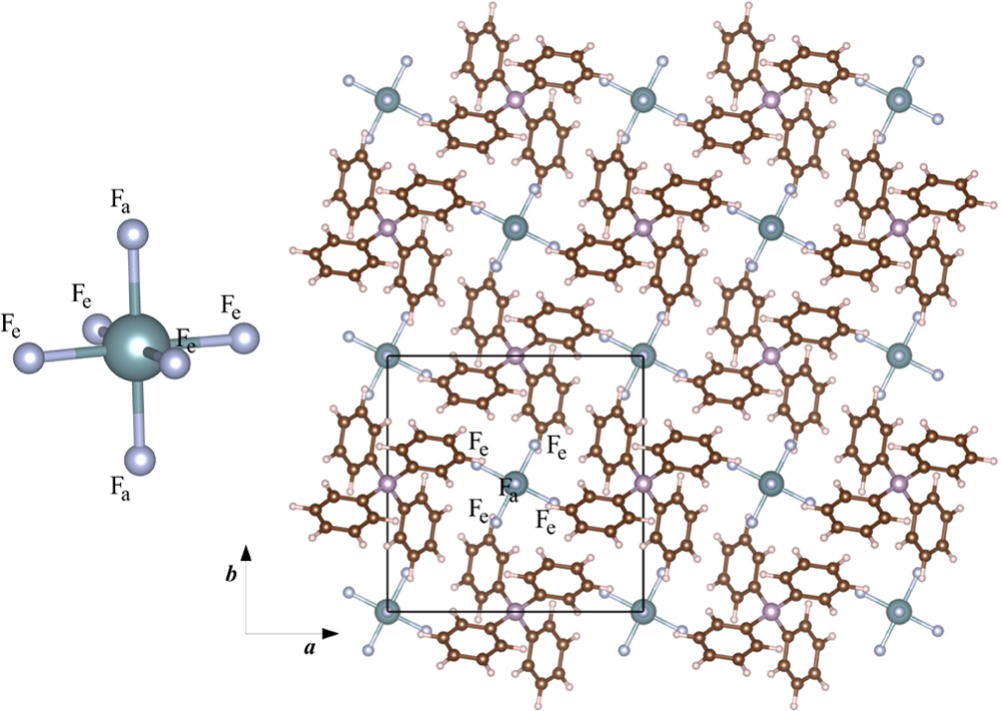
Atomic structure of the [PPh_4_^+^][UF_6_^–^] tetragonal crystal (view down the **c** crystallographic axis). The UF_6_ molecular fragments in the crystal are distorted with four equatorial fluorine atoms, F_e_, and two slightly more elongated apical fluorine atoms, F_a_.

Our analysis of chemical bonding starts from the inspection of the orbital shell populations and oxidation state of U in the four systems here considered (three molecules and one crystal), as reported in Table 1. The 32 outermost valence electrons of U are explicitly treated in the calculations (atomic electronic configuration: 5*s*^2^ 5*p*^6^ 5*d*^10^ 5*f*^3^ 6*s*^2^ 6*p*^6^ 6*d*^1^ 7*s*^2^). Atomic charges are computed from a simple Mulliken approach as well as from QTAIMAC. While Mulliken atomic charges are systematically smaller than Bader ones, trends along the series of four systems are quite consistent in the two cases. According to the QTAIMAC, the atomic charges of U and F are of +3.48 and −0.58 in s-UF_6_. Orbital shell populations reveal that the 7*s*^2^ electrons of U are transferred to the 2*p* orbitals of F, along with one of the three *f* electrons in 5*f*^3^. The populations of *d*-type orbitals appear to be less affected by bonding but show a clear trend from the neutral to charged species. Table 1 also shows how *g*-type functions (unpopulated on the isolated U atom) are partially involved in the description of the U–F bonds, with a population of 0.02 electrons. In this respect, we stress that by working in terms of spherical and not Cartesian functions, our *g*-type functions are not contaminated by *s*-type character.

**Table 1:** Mulliken Populations of Orbital Shells of the U Atom in the Four Systems Here Considered^a^

	s-UF6	s-UF_6_^–^	a-UF_6_^–^	cry-UF_6_^–^
populations				
s	–1.849	–1.877	–1.875	–1.868
p	–0.144	–0.099	–0.114	–0.118
d	0.051	–0.101	–0.096	–0.086
f	–1.069	–0.815	–0.763	–0.765
g	0.023	0.016	0.017	0.016
atomic charge				
*q*_U_^M^	2.987	2.877	2.831	2.819
*q*_U_^B^	3.477	3.218	3.217	3.212

a Differences with respect to the neutral atomic configuration 5*s*^2^ 5*p*^6^ 5*d*^10^ 5*f*^3^ 6*s*^2^ 6*p*^6^ 6*d*^1^ 7*s*^2^ are reported. Mulliken and Bader atomic charges of *U* are reported in the last two rows of the table. Bader’s charges are obtained through the QTAIMAC by numerical integration of the electron density over the U atomic basin. Results obtained at the B3LYP/BSA level.

Passing from the neutral species (s-UF_6_) to the anion (s-UF_6_^–^), the positive charge of U decreases to +3.22 and the negative charge of F becomes −0.70. This shows that about 70% of the extra electron is hosted by 2*p* orbitals of the F atoms and less than 30% by the central U atom. In particular, in the charged species, the 5*f*^3^ orbitals of U get less depopulated while 6*d*^1^ orbitals get significantly more depopulated. The distortion of the charged species induced by the crystal field, with the formation of two more elongated apical U–F_a_ bonds and four shorter U–F_e_ equatorial bonds, produces an overall decrease in the absolute value of the atomic charges of U and F, thus suggesting a lower ionicity and a larger degree of covalency of the bonds. This is already seen in passing from s-UF_6_^–^ to a-UF_6_^–^ and becomes even more pronounced when the effect of intermolecular interactions on the electron distribution of the molecule are explicitly taken into account in the crystalline environment (cry-UF_6_^–^). We will get back to this point later when various bond type descriptors from the QTAIMAC will be presented and discussed.

The crystalline environment of the uranium hexafluoride species in [PPh_4_^+^][UF_6_^–^] induces its geometrical frustration from a symmetric octahedron to a distorted one with two symmetry-independent sets of fluorine atoms (two apical F_a_ and four equatorial F_e_), which is also reflected in its electronic structure. This structural distortion is larger in the experimental than in the optimized theoretical structure. Figure 2 reports the atomic charges of F atoms, as obtained from QTAIMAC by numerical integration of the electron density over the corresponding atomic basins, for the three ionic species here considered (s-UF_6_^–^, a-UF_6_^–^, and cry-UF_6_^–^). In the symmetric species, the atomic charge of the six equivalent F atoms is −0.703. In the distorted molecular fragment (as extracted from the crystal) we observe a splitting of the atomic charges of the F atoms, with a larger charge in the apical atoms and a lower charge in the equatorial ones. This trend is confirmed when going further from the molecular fragment to the actual crystal, with an enhancement of the splitting. In particular, crystal field effects are such to increase the charge of the two apical F atoms, which is consistent with the experimental evidence of a larger deformation density on the apical atoms.^31^ Inspection of Figure 2 thus suggests a higher ionicity (*i.e.*, lower covalency) in the two apical U–F_a_ bonds than in the four equatorial U–F_e_ bonds. This evidence will further be corroborated below by the analysis of various bond descriptors from the QTAIMAC and, moreover, will prove crucial in the assessment of the reliability of different models used in the reconstruction of the experimental density.

**Figure 2:**
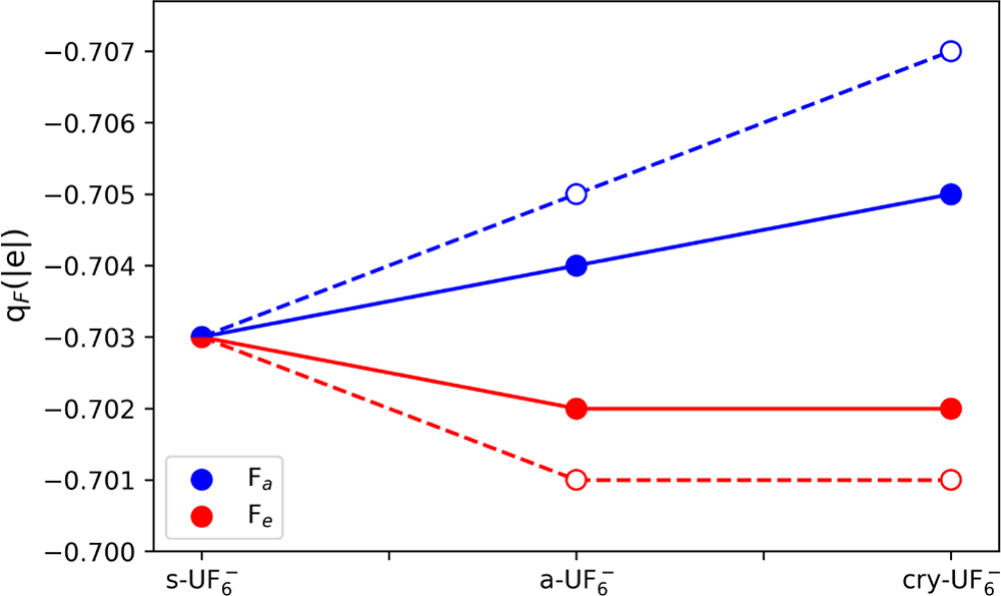
Atomic charges of the F atoms in the three ionic species here considered (s-UF_6_^–^, a-UF_6_^–^, and cry-UF_6_^–^) as obtained from QTAIMAC at the B3LYP/BSA level. Filled symbols and continuous lines refer to the optimized structures, and empty symbols and dashed lines refer to the experimental structure.

Let us now analyze the chemical bonding of the UF_6_^–^ species in the [PPh_4_^+^][UF_6_^–^] crystal more closely. We start by performing a topological analysis of the electron density ρ(**r**), which allows us to find and characterize bond critical points along the U–F_a_ and U–F_e_ bonds. Table 2 reports several bond descriptors evaluated at the bond critical points from the QTAIMAC. Computed values for a-UF_6_^–^ and cry-UF_6_^–^ are reported and compared with experimental values obtained from two different models in ref (31) (referred to as models 1b and 1c). The overall agreement between computed and experimental values is remarkable, both clearly confirming the mixed ionic/covalent nature of the U–F bonds based on the various descriptors with ∇^2^ρ > 0, *H* < 0, 1 < |*V*|/*G* < 2, and small and negative *H*/ρ at the bond critical points. From a quantitative point of view, the agreement is particularly impressive on ρ and |*V*|/*G*. Indeed, the computed values for the electron density at the bond critical points fall between the two values obtained experimentally from the two models: ρ_1b_^exp^ < ρ^calc^ < ρ_1c_^exp^, with deviations never exceeding 4% and often below 1%. The |*V*|/*G* ratio at the critical points is about 1.3 in all cases, with small deviations between theory and experiment.

**Table 2:** Descriptors of Chemical Bonding from the QTAIMAC of the Distorted UF_6_^–^ in the [PPh_4_^+^][UF_6_^–^] Crystal^a^

	calculated (this study)									experimental (ref ^31^)					
	a-UF_6_^–^			cry-UF_6_^–^			cry-eg-UF_6_^–^			model 1b			model 1c		
	F_e_	F_a_	Δ	F_e_	F_a_	Δ	F_e_	F_a_	Δ	F_e_	F_a_	Δ	F_e_	F_a_	Δ
*l*_U–F_ (Å)	2.076	2.082	0.006	2.076	2.082	0.006	2.065	2.077	0.012	2.065	2.077	0.012	2.065	2.077	0.012
*d*_U–CP_ (Å)	1.144	1.148	0.004	1.144	1.147	0.003	1.138	1.144	0.006	1.169	1.169	0.000	1.162	1.166	0.004
ρ (*e*/Å^3^)	0.871	0.864	–0.007	0.874	0.862	–0.013	0.897	0.871	–0.026	0.868	0.827	–0.041	0.881	0.885	0.004
∇^2^ρ (*e*/Å^5^)	10.314	10.242	–0.072	10.300	10.248	–0.052	10.505	10.395	–0.110	11.016	11.643	0.627	10.545	9.023	–1.522
|*V*|/*G*	1.318	1.312	–0.006	1.320	1.313	–0.007	1.329	1.315	–0.013	1.329	1.278	–0.051	1.322	1.413	0.091
*H*/ρ (a.u.)	–0.386	–0.378	0.008	–0.387	–0.379	0.008	–0.401	–0.385	0.017	–0.436	–0.380	0.056	–0.418	–0.503	–0.085

a Bond length, *l*_U–F_; distance between U and the bond critical point, *d*_U–CP_; values of several local quantities at the bond critical point, such as the electron density (ρ), the Laplacian of the density (∇^2^ρ), the ratio between the potential energy density and kinetic energy density (|*V*|/*G*), and the bond degree (*H*/ρ) (*i.e.*, ratio between total energy density and electron density). Values are reported for the two bonds U–F_a_ and U–F_e_. The difference of each quantity between the two bonds Δ = U–F_a_ – U–F_e_ is also reported. Computed values at the B3LYP/BSA level (this study) for a-UF_6_^–^ and cry-UF_6_^–^ are reported and compared with experimental values of the crystal as obtained from two different models from ref (31). Results from calculations performed on the experimental geometry of the crystal are also reported (cry-eg-UF_6_^–^).

Let us now address a subtle (and critical) aspect of the chemical bonding of the system, that is, the difference in bonding of U–F_a_ and U–F_e_. Comparison of a-UF_6_^–^ and cry-UF_6_^–^ results in Table 2 shows how the electron density at the bond critical point is significantly affected by the intermolecular interactions. In particular, the difference Δρ between the apical and equatorial bonds increases almost by a factor of 2 in passing from a-UF_6_^–^ to cry-UF_6_^–^. Inspection of the computed bond descriptors confirms the larger covalent character of the equatorial bonds that are indeed characterized by a shorter bond length, larger value of the density, larger value of |*V*|/*G*, and a more negative value of the bond degree *H*/ρ. Comparison with the experiment is much more critical because, on this subtle aspect, the two models 1b and 1c are in qualitative disagreement, with model 1b describing equatorial bonds slightly more covalent than apical ones (matching the theoretical predictions) but model 1c describing apical bonds as more covalent than equatorial ones. On the one hand, model 1c allowed for a more stable refinement;^31^ on the other hand, the shorter equatorial bonds in the structure would seem consistent with their higher degree of covalency as described by model 1b and by present quantum-mechanical calculations.

We now analyze the topology of the Laplacian of the density ∇^2^ρ(**r**), which provides additional information on the spatial distribution of the electrons and in particular on the asphericity of (bonded) atoms.^58^ Critical points of the Laplacian correspond to charge concentrations and depletions in the core and valence shells. Valence shell charge concentrations (VSCCs) are particularly relevant to the rationalization of chemical bonding and can be analyzed in terms of critical points of the Laplacian of type (3, + 3), *i.e.*, minima. For light atoms, they often correspond to bonding and lone pair regions. Their interpretation becomes progressively more complex as one moves to heavier metals. In the context of metal-containing molecules and complexes, for instance, several *d*^6^ transition metal compounds (*O*_*h*_ structure) are characterized by 8 VSCCs arranged at the vertices of a cube, with metal–ligand axes passing through the center of the cube faces.^59,60^ These critical points can still be easily interpreted by the ligand-field theory as those regions in space where charge concentration of the metal is favored by a lower repulsion with the electrons of the ligands. The situation is expected to become more articulated when passing to actinides, whose valence involves different principal and angular quantum numbers. Previous theoretical calculations^31^ on the molecular fragment UF_6_^–^ extracted from the [PPh_4_^+^][UF_6_^–^] crystal predicted a qualitatively similar spatial distribution of the VSCCs around the U atom as in *d*^6^ transition metals (see panels in the last column of Figure 3c). However, the topology of the Laplacian derived by the experimental density of the crystal is significantly different, with both quantitative and qualitative discrepancies with respect to those first calculations, as shown in Figure 3. A total of 14 VSCCs were reported around the U atom: (i) 8 critical points arranged at the vertices of a cube with the edges slightly tilted off the U–F axes (red spheres in the figure); (ii) 4 critical points forming a square in the equatorial plane, with vertices slightly tilted off the bisector of the Fe–Û–Fe angle (yellow spheres in the figure); (iii) 2 critical points along the U–F_a_ axes (yellow spheres in the figure). Experimentally, all 14 VSCCs are at a distance of about 0.38 Å from U while in the previous calculations the 8 VSCCs were found at about 0.85 Å, which seems inconsistent with the radial distribution of the valence of U, as discussed below. Getting rid of such large discrepancies in the description of the topology of the Laplacian of the [PPh_4_^+^][UF_6_^–^] crystal around the U atom is therefore compelling to assess the accuracy of the experimental procedure as well as that of any theoretical approach in the description of the electron density of actinide compounds. The first panels in Figure 3c show the VSCC (3, +3) critical points of the Laplacian as obtained from present quantum-mechanical calculations on both the a-UF_6_^–^ and cry-UF_6_^–^ systems. Inspection of the figure suggests that the agreement with the experimental spatial distribution of the Laplacian is recovered to a large extent. Present calculations are indeed able to confirm the whole set of 14 critical points found in the experiments. The predicted radial distance of the (3, +3) critical points of the Laplacian is of 0.30 Å and coincides with the minimum of the VSCC of the principal quantum number 6 (the VSCC for *n* = 7 is not visible neither in the isolated U atom Laplacian profile, nor in the UF_6_ compound, as the negative Laplacian due to *n* = 7 orbital components is overcompensated by positive Laplacian contributions due to the innermost shells). As a consequence, only a VSCD (valence shell charge depletion) is visible after the *n* = 6 VSCC (see Figure 3 b). Furthermore, according to present calculations, the 14 critical points can be grouped into two independent sets with slightly different properties: 8 critical points arranged at the vertices of a cube (red spheres in the figure) and 6 critical points arranged at the vertices of an octahedron (yellow spheres in the figure). The only difference with respect to the experiment consists in the red cube and yellow octahedron not being tilted off the U–F bonds, which, however, seems consistent with the symmetry of the system. The spatial distribution of the two sets of VSCCs around the U atom can be rationalized in terms of the hybridization of the valence atomic orbitals. It has recently been shown that a *sp*^3^*d*^2^ hybridization leads to a octahedral 6-fold coordination and a *sp*^3^*d*^3^*f* hybridization leads to a cubic 8-fold coordination.^61^

**Figure 3:**
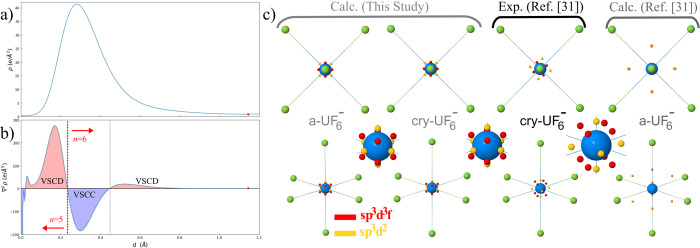
Topology of the Laplacian ∇^2^ρ(**r**) of the electron density of UF_6_^–^: (a) electron density profile along the U–F_e_ bond (the red circle denotes the location of the bond critical point); (b) Laplacian profile along the U–F_e_ bond (the dashed red vertical line separates the *n* = 5 from the *n* = 6 valence radial region); (c) spatial distribution of the VSCC critical points (3, +3) of the Laplacian around the U atom in present calculations, in the experiments, and in previous calculations. A zoomed-in view in the vicinity of the U atom is also provided for the first three data sets (*i.e.*, for present calculations and previous experiments).

In conclusion, we have extended the QTAIMAC implementation in the Topond package to *f*- and *g*-type basis functions. This now makes it possible to analyze the electron density of materials containing lanthanides and actinides, as obtained from LCAO quantum-mechanical calculations. Application of this methodology to the rationalization of chemical bonding in [PPh_4_^+^][UF_6_^–^] crystals nicely shows the potential of the approach. In particular, some previously reported discrepancies between experimental and theoretical features of the topology of the density are reconsidered and largely removed, which proves significant in the mutual validation of the experimental and theoretical route to the accurate description and analysis of the electron density of actinide compounds and materials.

## Supporting Information Available

Description of the basis sets used for the calculations; theoretical deformation densities (PDF)
