# Supplementary material for: Charge Density Analysis of Actinide Compounds from the Quantum Theory of Atoms in Molecules and Crystals
Source: J Phys Chem Lett. 2021 Feb 12;12(7):1862–8. doi: 10.1021/acs.jpclett.1c00100 (PMC8028320; doi:10.1021/acs.jpclett.1c00100)
Supplement: Supplementary file 1 — jz1c00100_si_001.pdf [file jz1c00100_si_001.pdf]

Supporting Information: Charge Density Analysis of Actinide Compounds from the Quantum Theory of Atoms in Molecules and Crystals

Alessandro Cossard,† Jacques K. Desmarais,† Silvia Casassa,† Carlo Gatti,‡ and Alessandro Erba*,†

†*Dipartimento di Chimica, Università di Torino, via Giuria 5, 10125 Torino, Italy*

‡*CNR-SCITEC, Istituto di Scienze e Tecnologie Chimiche "Giulio Natta", via C. Golgi 19, 20133 Milano, Italy*

E-mail: alessandro.erba@unito.it

1 Basis Sets BSA

1.1 For the U Atom

Basis set A is a ECP60MDF-(10s9p7d5f1g)/[10s9p7d5f1g] with a small-core pseudopotential describing the 60 innermost electrons of U. Both scalar and spin-orbit relativistic effects are accounted for in the definition of the pseudopotential.[S1] The numbers on the right of the label describe the valence part: we indicate within round brackets the number of Gaussian primitive functions used for the various angular quantum numbers and within square brackets the number of shells in which they are contracted. In this case, BSA is a fully uncontracted basis. With respect to the original basis set optimized for molecular calculations, some very diffuse exponents have been removed (crucially the most diffuse *p*-type exponent) that were causing linear dependencies in the periodic calculations. The basis set is explicitly reported below in the Crystal input format:

```
292 31
INPUT
32. 0 4 8 8 8 6
16.91870874 529.53526911 0
3.40970576 4.27018845 0
0.79302733 0.09998874 0
0.19378381 0.00626781 0
13.16953414 100.93359134 0
10.60784728 175.95423897 0
2.69049397 -0.00210787 0
2.08929800 -0.19041648 0
0.54050990 0.00494627 0
0.40482776 -0.01652483 0
0.11250285 0.00082033 0
0.09508873 -0.00100028 0
9.06784123 62.85927902 0
```

```
8.53362678 90.20882494 0
1.63646790 -0.08282418 0
1.54425719 -0.15307917 0
0.47961552 -0.00008720 0
0.41164502 0.00484078 0
0.13990510 -0.00006136 0
0.17494682 -0.00240839 0
5.14746012 15.68628229 0
5.29241394 22.32105345 0
1.05726701 -0.20689333 0
0.98063114 -0.08434451 0
0.48259555 0.06084446 0
0.55434882 0.00231264 0
0.23674544 -0.00204069 0
0.21559852 0.00348388 0
18.83643086 -44.41029420 0
18.74850924 -53.65339478 0
6.49279545 -2.55219343 0
6.57472519 -3.34380088 0
2.58151924 0.04527524 0
2.58690949 0.05637947 0
0 0 1 2 1
  29520.834 1.
0 0 1 2 1
  4449.8874 1.
0 0 1 2. 1
  1018.7754 1.
0 0 1 0 1
  289.5348 1.
```

```
0 0 1 0 1
  46.9990 1.
0 0 1 0 1
  23.9791 1.
0 0 1 0 1
  10.1441 1.
0 0 1 0 1
  2.7658 1.
0 0 1 0 1
  0.6151 1.
0 0 1 0 1
  0.2780 1.
0 2 1 6 1
  499.7488 1.
0 2 1 6 1
  114.0192 1.
0 2 1 0 1
  15.6492 1.
0 2 1 0 1
  7.9843 1.
0 2 1 0 1
  3.1325 1.
0 2 1 0 1
  1.6077 1.
0 2 1 0 1
  0.7061 1.
0 2 1 0 1
  0.3229 1.
0 3 1 10 1
```

```
  75.1703 1.
0 3 1 1 1
  20.7869 1.
0 3 1 0 1
  5.5167 1.
0 3 1 0 1
  2.6058 1.
0 3 1 0 1
  1.2781 1.
0 3 1 0 1
  0.5617 1.
0 3 1 0 1
  0.2135 1.
0 4 1 3. 1
  8.1761 1.
0 4 1 0 1
  3.5111 1.
0 4 1 0 1
  1.6789 1.
0 4 1 0 1
  0.7604 1.
0 4 1 0 1
  0.3170 1.
0 5 1 0 1
  0.7604 1
```

1.2 For the F Atoms

Basis set A for F atoms is an all-electron one: (23s9p2d2f)/[5s5p2d2f]. The basis set is explicitly reported below in the CRYSTAL input format:

```
9 14
0 0 10 2. 1.00
      1.950000D+04           5.070000D-04
      2.923000D+03           3.923000D-03
      6.645000D+02           2.020000D-02
      1.875000D+02           7.901000D-02
      6.062000D+01           2.304390D-01
      2.142000D+01           4.328720D-01
      7.950000D+00           3.499640D-01
      2.257000D+00           4.323300D-02
      8.815000D-01          -7.892000D-03
      3.041000D-01           2.384000D-03
0 0 10 2. 1.00
      1.950000D+04          -1.170000D-04
      2.923000D+03          -9.120000D-04
      6.645000D+02          -4.717000D-03
      1.875000D+02          -1.908600D-02
      6.062000D+01          -5.965500D-02
      2.142000D+01          -1.400100D-01
      7.950000D+00          -1.767820D-01
      2.257000D+00           1.716250D-01
      8.815000D-01           6.050430D-01
      3.041000D-01           3.695120D-01
0 0 1  0. 1.00
      2.257000D+00           1.000000D+00
0 0 1  0. 1.00
      3.041000D-01           1.000000D+00
0 0 1  0. 1.00
      0.0915800              1.0000000
```

```
0 2 5  5.0 1.00
      4.388000D+01          1.666500D-02
      9.926000D+00          1.044720D-01
      2.930000D+00          3.172600D-01
      9.132000D-01          4.873430D-01
      2.672000D-01          3.346040D-01
0 2 1  0. 1.00
      9.132000D-01          1.000000D+00
0 2 1  0. 1.00
      2.672000D-01          1.000000D+00
0 2 1  0. 1.00
      0.0736100             1.0000000
0 2 1  0. 1.00
      3.107000D+00          1.000000D+00
0 3 1  0. 1.00
      8.550000D-01          1.000000D+00
0 3 1  0. 1.00
      0.2920000             1.0000000
0 4 1  0. 1.00
      1.917000D+00          1.0000000
0 4 1  0. 1.00
      0.7240000             1.0000000
```

1.3 For H, C and P Atoms

The pob-TZVP-def2 basis sets were used for H, C and P atoms.[S2]

2 Theoretical Deformation Densities

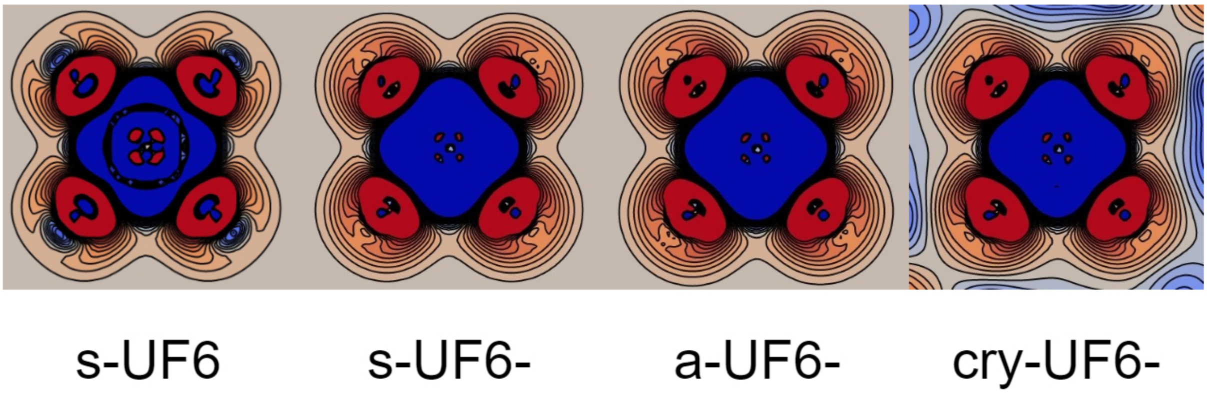

Figure S1: Deformation density iso-valued contour maps. The minimum value is -0.01 e/bohr^3, the maximum value is 0.01 e/bohr^3 and the step between iso-valued lines is 0.001 e/bohr^3.

References

(S1) Dolg, M.; Cao, X. Accurate relativistic small-core pseudopotentials for actinides. Energy adjustment for uranium and first applications to uranium hydride. *J. Phys. Chem. A* **2009**, *113*, 12573–12581.

(S2) Vilela Oliveira, D.; Laun, J.; Peintinger, M. F.; Bredow, T. BSSE-correction scheme for consistent gaussian basis sets of double-and triple-zeta valence with polarization quality for solid-state calculations. *J. Comput. Chem.* **2019**, *40*, 2364–2376.
